# Interventions to prevent post-tuberculosis sequelae: a systematic review and meta-analysis

**DOI:** 10.1016/j.eclinm.2024.102511

**Published:** 2024-02-26

**Authors:** Kefyalew Addis Alene, Lucas Hertzog, Beth Gilmour, Archie C.A. Clements, Megan B. Murray

**Affiliations:** a School of Population Health, Faculty of Health Sciences, Curtin University, Australia; b Geospatial and Tuberculosis Research Team, Telethon Kids Institute, Australia; c Department of Global Health and Social Medicine, Harvard Medical School, Boston, MA, USA; d Peninsula Medical School, University of Plymouth, Plymouth, United Kingdom

**Keywords:** Tuberculosis, Sequelae, Prevention, Intervention, Systematic review, Meta-analysis

## Abstract

**Background:**

Tuberculosis (TB) remains a global public health challenge, causing substantial mortality and morbidity. While TB treatment has made significant progress, it often leaves survivors with post-TB sequelae, resulting in long-term health issues. Current healthcare systems and guidelines lack comprehensive strategies to address post-TB sequelae, primarily due to insufficient evidence. This systematic review and meta-analysis aimed to identify effective interventions for preventing post-TB sequelae.

**Methods:**

A systematic search was conducted across four databases including PubMed, SCOPUS, Web of Science, and Cochrane Central Register of Controlled Trials from inception to September 22, 2023. Eligible studies reported interventions designed to prevent post-TB sequelae were included. A random effect meta-analysis was conducted where applicable, and heterogeneity between studies was evaluated visually using forest plots and quantitatively using an index of heterogeneity (I^2^). This study is registered with PROSPERO (CRD42023464392).

**Findings:**

From the 2525 unique records screened, 25 studies involving 10,592 participants were included. Different interventions were evaluated for different outcomes. However, only a few interventions were effective in preventing post-TB sequelae. Rehabilitation programs significantly improved lung function (Hedges's g = 0.21; 95% confidence interval (CI): 0.03, 0.39) and prevented neurological sequelae (relative risk (RR) = 0.10; 95% CI: 0.02, 0.42). Comprehensive interventions and cognitive-behavioural therapy significantly reduced the risk of mental health disorders among TB survivors (Hedges's g = −1.89; 95% CI: −3.77, −0.01). In contrast, interventions targeting post-TB liver sequelae, such as vitamin A and vitamin D supplementation and hepatoprotective agents, did not show significant reductions in sequelae (RR = 0.90; 95% CI: 0.52, 1.57). Moreover, adjunctive therapies did not show a significant effect in preventing post-TB neurological sequelae (RR = 0.62, 95% CI: 0.31, 1.24).

**Interpretation:**

Rehabilitation programs prevented post-TB lung, neurologic and mental health sequelae, while adjuvant therapies and other interventions require further investigation.

**Funding:**

Healy Medical Research Raine Foundation, Curtin School of Population Health and the Australian National Health and Medical Research Council.

**Research in context**

**Evidence before this study**

There is a growing body of evidence indicating that tuberculosis (TB) survivors often experience a range of post-TB sequelae, resulting from the disease itself and the side effects of TB treatment. We searched PubMed, Cochrane Central, SCOPUS, and Web of Science databases from the inception of each database to 22 September 2023, for papers published in English, using terms related to TB, sequelae, and prevention. Multiple systematic reviews were found through our search, examining the prevalence and impact of post-TB sequelae and highlighting the substantial burden faced by TB survivors. Previous individual studies have also explored various interventions to mitigate these sequelae, including pulmonary rehabilitation programs, adjunctive therapies, nutritional supplementation, and psychological support. However, the evidence on the effectiveness of these interventions is limited, and there is a lack of comprehensive systematic reviews that synthesise the available evidence across different domains of post-TB sequelae.

**Added value of this study**

This comprehensive systematic review and meta-analysis identifies effective interventions for preventing a wide range of post-TB sequelae, including lung, liver, neurologic, and mental health sequelae. The results show the importance of timing for specific interventions in addressing different sequelae. Rehabilitation programs were effective when implemented during treatment in preventing post-TB lung and neurologic sequelae as well as mental health disorders, whereas the effectiveness of adjuvant therapies and other interventions requires further investigation. This comprehensive analysis fills a crucial knowledge gap and helps inform evidence-based strategies for post-TB care and management.

**Implications of all the available evidence**

Existing evidence shows the importance of considering the long-term health of TB survivors. Integrating pulmonary rehabilitation programs during TB treatment can substantially improve lung function and prevent other types of post-TB sequelae. However, further research and innovation are required, particularly in preventing post-TB liver sequelae arising from drug-induced liver injury (DILI), where existing interventions show limited efficacy. The findings of current evidence can also inform healthcare providers and policymakers about the importance of post-TB care and the potential benefits of specific interventions. Expanding the remit of current TB programs to include strategies for preventing post-TB sequelae, such as pulmonary rehabilitation programs, will help reduce the morbidity and suffering associated with this disease.

## Introduction

Tuberculosis (TB) is a serious public health concern and the leading cause of death from an infectious disease, killing a total of 1.3 million people in 2022.^1^ An estimated 10.6 million people develop TB disease every year, with around 70% receiving treatment.^1^ While substantial progress has been achieved with an estimated 66 million lives saved through TB diagnosis and treatment over the past two decades,^1^ people who survive TB face a considerable and under-recognised burden of ongoing morbidity including physical impairment and psychosocial problems even after completion of treatment (i.e. collectively known as post-TB sequelae).^2^^,^^3^ While post-TB lung disease refers to consequences associated with pulmonary TB, post-TB sequelae are broader, encompassing sequelae that affect various organ systems and parts of the body beyond the pulmonary region.^4^ These post-TB sequelae mainly arise from the disease process or the side effects related to TB treatment and increase the risk of permanent disability, premature mortality, ongoing stigma, and poor quality of life.^5^ Previous systematic reviews on the global burden of TB-related disability showed that the prevalence of respiratory function impairment among TB survivors ranges from 15% to 60%.^2^^,^^6^ A modelling study has shown that nearly half of the global burden of morbidity and mortality related to TB is attributed to post-TB lung diseases.^7^

As the number of TB survivors is expected to rise in the future due to improved diagnosis and treatment, there is an urgent need to focus on post-treatment care. However, current healthcare systems and TB programs do not generally focus attention on people affected by TB after treatment completion. For example, the current World Health Organization (WHO) guidelines for TB management define ‘treatment success’ using microbiological outcomes and survival only, and measures of TB-associated morbidity remain solely focused on the period prior to and during treatment.^8^ The recent WHO policy brief on TB-associated disability recognizes post-TB sequelae as a substantial challenge in TB programs. However, it neglects post-TB care and lacks strategies for following up with TB survivors after completing their treatment.^9^ Moreover, the recent United Nations New York declaration on TB has neglected to address the issue of post-TB sequelae.^10^ The reason why current TB guidelines and policy documents omit consideration of post-TB sequelae is partly due to a lack of comprehensive evidence.

While several systematic reviews have been conducted to identify strategies for improving end-of-treatment outcomes, there remains a critical research gap in identifying preventive strategies for mitigating the post-TB treatment sequelae burden. The limited availability of observational studies or clinical trials has yielded inconclusive evidence. This systematic review and meta-analysis aims to provide comprehensive evidence on the effectiveness of existing interventions in preventing post-TB sequelae.

## Methods

### Search strategy

This systematic review and meta-analysis was conducted following the updated guideline for reporting systematic reviews (Table S1 and Table S2).^11^ The study protocol has been registered at the PROSPERO international registry for systematic reviews [CRD42023464392]. Systematic literature searches were conducted in PubMed, Cochrane Central, SCOPUS, and Web of Science databases from the inception of each database to 22 September 2023. The Medical Subject Headings (MeSH) term and a combination of keywords related to TB, sequelae, and prevention were used for the search. We systematically conducted searches for key terms across titles and abstracts. A complete search strategy for each database is available in the supplementary file (Table S3). The reference lists and citations of the retrieved articles were checked manually for additional studies. The authors of the papers were contacted through email when there was a need for additional information.

### Eligibility criteria

The selection criteria to identify potential studies were based on the PICOS (Population, Intervention, Control, Outcome and Study type) criteria. Interventional and observational studies conducted on adults or children with TB were included in the systematic review if they reported relevant information about the effectiveness of interventions in preventing post-TB sequelae. The main outcome of interest was a reduction in post-TB sequelae, which was broadly defined as any clinical condition that TB survivors experience after and as a consequence of TB illness or TB medication.^5^ These include any long-term sequelae such as pulmonary, liver, neurologic, visual, hearing, and cardiac sequelae as well as mental health disorders that occur as a result of TB illness or side effects of TB medications.^12^

We excluded studies that reported temporary adverse events during treatment such as hepatotoxicity and renal toxicity that was resolved by a change of medication. We also excluded correspondence, reviews, editorials, and conference abstracts without sufficient information on the main outcome of interest. Studies conducted on animals and non-English language articles were also excluded. When multiple studies used the same data, we included the study with the most detailed clinical data, with the largest sample size or with the longest follow-up period to avoid duplication. Detailed information about the inclusion and exclusion criteria based on the PICOS principle is available in the supplementary information (Table S4).

### Procedures

All citations identified through our search strategy were imported into EndNote library version X8 (Thompson Reuters, New York, NY, USA). After removing duplicates, articles were uploaded into Rayyan^13^ for screening. Two authors (KAA and BG) independently screened the titles and abstracts of studies and reviewed full-text articles to identify eligible studies, ensuring a blinded review process between the two reviewers. Any discrepancies were discussed and resolved by consensus.

Data were extracted from included articles using a piloted form by the same two researchers (KAA and BG). We collected information about the characteristics of patient cohorts, studies, and outcomes of interest, and recorded this information in an MS Excel spreadsheet. Comprehensive details on the extracted variables from included studies can be found in the supplementary information (Table S5).

The quality of the included studies and the risk of bias were assessed by the same two researchers (KAA and BG) using the revised Cochrane Risk of Bias Tool for interventional studies^14^ and Newcastle–Ottawa Quality Assessment for observational studies.^15^ The Cochrane methodology assessed each study with the following five domains: bias arising from (I) the randomisation process; (II) deviations from interventions; (III) missing the outcome data; (IV) measurement of outcome; and (V) selection of the reported result. Each domain had specific signalling questions and responses to these questions led to the judgment of “low risk of bias”, “some concerns” and “high risk of bias”. Therefore, a study with a low risk of bias for all domains and some concern for at least one domain was judged to have “low risk of bias” and “some concerns” respectively. A study with a high risk of bias for at least one domain or that was judged as having some concerns in multiple domains in a way that substantially affected the results was judged to have a “high risk of bias”. The modified Newcastle-Ottawa Quality Assessment Scale^16^ used to assess observational studies had scores ranging from zero to nine with scores 1 to 4, 5 to 7, and 8 to 9 representing low, medium and high-quality groupings, respectively.

### Statistical analysis

Narrative syntheses were conducted using all included studies to describe the outcomes of the studies. Additionally, meta-analyses were performed separately for each outcome only when two or more studies were available on the outcome of interest. The effectiveness of interventions on the outcome of interest (in preventing post-TB sequelae) was assessed using pooled relative risk (RR) with 95% confidence intervals (CIs) for dichotomous outcomes (i.e. normal vs abnormal liver and neurologic function). A RR and corresponding 95% CI were computed using the total numbers of participants and events in each arm within each study. For continuous variables such as lung function tests and mental health disorder scores (i.e., anxiety and depression scores), Hedges's g with 95% CIs was utilized. Hedges's g was selected over standardized mean differences due to its appropriateness for handling limited sample sizes, incorporating a correction factor to account for potential overestimation of the effect size in studies with small numbers of participants. Heterogeneity between studies was assessed visually by forest plots and quantitatively by the index of heterogeneity squared (I^2^) statistics, with 95% CI. The I^2^ statistic measures the proportion of observed variance between studies that is not due to chance (rather due to real differences across study populations and interventions). An I^2^ value greater than 75% was interpreted as evidence of a substantial level of heterogeneity.^17^ Sensitivity analyses were conducted using fixed effects meta-analyses for endpoints with little evidence of heterogeneity. Sub-group analysis was conducted to explore the source of heterogeneity. The risk of publication bias was assessed by examining funnel plot symmetry and by conducting Egger's regression test where A significant P-value <0.05 indicates publication bias. Adjusted variables for the observational studies are available in Table S6. The data analysis was conducted by Stata Software version 17.

### Ethics

The present study did not require informed consent or approval by the local Ethics Committees, due to the nature of the research.

### Role of the funding source

The funder of the study had no role in study design, data collection, data analysis, data interpretation, or writing of the report.

## Results

**Fig. 1:**
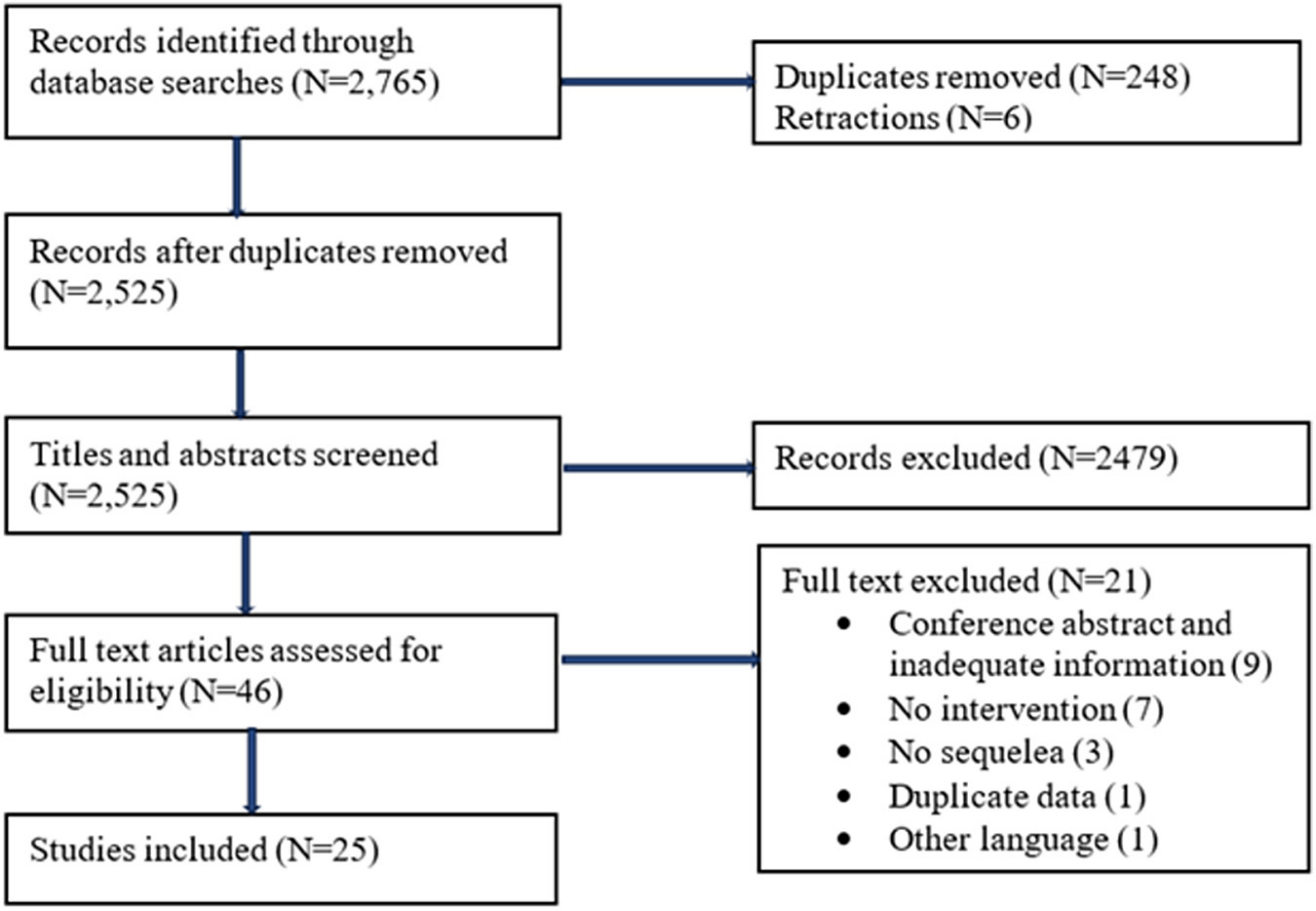
Flow chart showing literature search and study selection.

Our systematic search identified a total of 2765 articles. After removing 248 duplicates and 6 retracted papers, the remaining 2525 articles were screened by title and abstract, leaving 46 articles for full-text review. Finally, 25 articles fulfilling the eligibility criteria were included in this systematic review and meta-analysis, representing a total of 10,592 study participants (Fig. 1). The reasons for exclusion are provided in the supplementary files (Table S7).

### Study characteristics

Table 1 provides detailed characteristics of the included studies. The studies were conducted between 1983 and 2023 in 12 different countries. The majority of studies were conducted in China (n = 7), South Africa (n = 5), and India (n = 4). The sample size of the included studies varied from 29 to 6743 (median 86). Most studies assessed possible interventions for preventing post-TB lung function sequelae (n = 9), liver function sequelae (n = 6), neurological sequelae (n = 5), and mental health disorders (n = 4). A few studies also assessed interventions for preventing hearing sequelae (n = 2), visual sequelae (n = 2), and cardiac sequelae (n = 1). Several possible interventions were investigated in the existing literature for their effectiveness in preventing post-TB sequela. These interventions include pulmonary rehabilitation programs, adjuvant therapies, comprehensive nursing care, micronutrient supplementation, hepatoprotective agents, cognitive behavioural therapy, and early medical interventions (Table 2).

**Table 1:** Characteristics of studies included in the systematic review and meta-analyses.

First author	Publication year	Country	Study design	Mean/median age (year)	Sample size	Intervention	Control	Length of intervention	Type of post-TB sequelae
Strange JIG	1988	South Africa	RCT	NA	240	Prednisolone	Placebo	11 weeks	Cardiac function
Ando M	2003	Japan	RCT	71	32	Pulmonary rehabilitation	Before intervention	9 weeks	Lung function
Nas K	2004	Turkey	Prospective cohort	37.9	47	Rehabilitation program	Before intervention	6 months	Neurologic
Torok MS	2011	Vietnam	RCT	35	545	Dexamethasone	Placebo	6–8 weeks	Neurologic
Grass DD	2014	South Africa	RCT	NA	67	Pulmonary rehabilitation	No intervention	6 weeks	Lung function
Jones R	2017	Uganda	RCT	45	34	Pulmonary rehabilitation	Before intervention	6 weeks	Lung function
Singh SM	2018	India	Prospective cohort	48	29	Pulmonary rehabilitation	Before intervention	8 weeks	Lung function
Li X	2019	China	RCT	72	183	Comprehensive care	Routine care	6 months	Anxiety & depression
Vashakidze SA	2019	USA	Cross-sectional	31	54	Surgical resection	No surgery	NA	Lung function
Shangase KK	2019	South Africa	Retrospective	33.3	86	Early medical intervention	No intervention	6 months	Hearing
Xiong K	2021	China	RCT	45	753	Vitamins A & D	No supplement	2 months	Liver function
Xu Z	2021	China	RCT	NA	150	Comprehensive intervention	Routine care	NA	Pulmonary depression & anxiety
Chen Q	2022	China	Retrospective cohort	47.1	6743	Silymarin/glycyrrhetinic acid	No intervention	1–30 days	Liver function
Manesh A	2023	India	Retrospective cohort	NA	90	Infliximab	No infliximab	≥1 dose	Neurological
Orooj M	2023	India	RCT	31.1	90	Pulmonary rehabilitation	No intervention	4 weeks	Pulmonary & mental health
Ahmed S	2020	India	RCT	25.5	62	Pulmonary rehabilitation	No intervention	12 weeks	Lung function
Visca D	2019	Italy	Retrospective cohort	75	43	Pulmonary rehabilitation	No intervention	3 weeks	Lung function
Zuo X	2022	China	RCT	41	45	Cognitive behavioural therapy	Routine care	2 months	Depression & Anxiety
Girgis NI	1983	Egypt	RCT	14	55	Dexamethasone	No steroids	2 weeks	Vision
Gu J	2015	China	RCT	36	568	Silibinin	No Silibinin	8 weeks	Liver function
Hakimizad R	2021	Iran	RCT	53	87	Acetyl-l-carnitine, alpha-lipoic acid & coenzyme Q10	Placebo	2 weeks	Liver function
Luangchosiri C	2015	Thailand	RCT	54	55	Silybum marinums	Placebo	4 weeks	Liver function
Schoeman JF	1997	South Africa	RCT	3	117	Prednisone	No steroid	1 month	Neurologic, hearing & vision
Schoeman JF	2003	South Africa	RCT	52	47	Thalidomide	Placebo	1 month	Neurologic
Zhang S	2015	China	RCT	NA	370	*Silybum marianum*	Vitamin C tablet	6 months	Liver function

**Table 2:** Number of studies reporting post-tuberculosis sequela and possible intervention strategies.

Post-TB sequelae	Number of studies	Intervention strategies for preventing sequelae
Lung function	9	• Pulmonary rehabilitation program • Adjunctive surgical resection • Comprehensive nursing care
Liver function	6	• Micronutrient supplementation (e.g., vitamin A and Vitamin D) • Mitochondrial nutrients (e.g., acetyl-l-carnitine, alpha-lipoic acid, and coenzyme Q10) • Hepatoprotective agents (e.g., silymarin, glycyrrhetinic acid, silibinin, and *Silybum marianum*)
Neurologic	5	• Adjunctive therapy (e.g., dexamethasone, prednisone, thalidomide, and infliximab) • Rehabilitation programs.
Mental health disorder	4	• Comprehensive interventions • Pulmonary rehabilitation programs • Cognitive-behavioural therapy
Hearing	2	• Early medical intervention • Adjuvant steroid therapy
Vision	2	• Adjuvant steroid therapy
Cardiac function	1	• Adjuvant steroid therapy

### Quality assessment

Twenty-four studies were evaluated using the Cochrane risk of bias assessment tool, resulting in the classification of 17 studies as “low risk”, four as “some concerns”, and three as “high risk” of bias. Among the studies classified as “high risk”, all had a bias in the cohort selection and one study also had the potential for bias in the matching of exposed and unexposed cohorts. One study evaluated using the adapted Newcastle Ottawa scale was classified as medium quality. The assessment scores for each of the included articles are available in the supplementary file (Table S8 and Table S9).

### Intervention to prevent post-TB lung sequelae

**Fig. 2:**
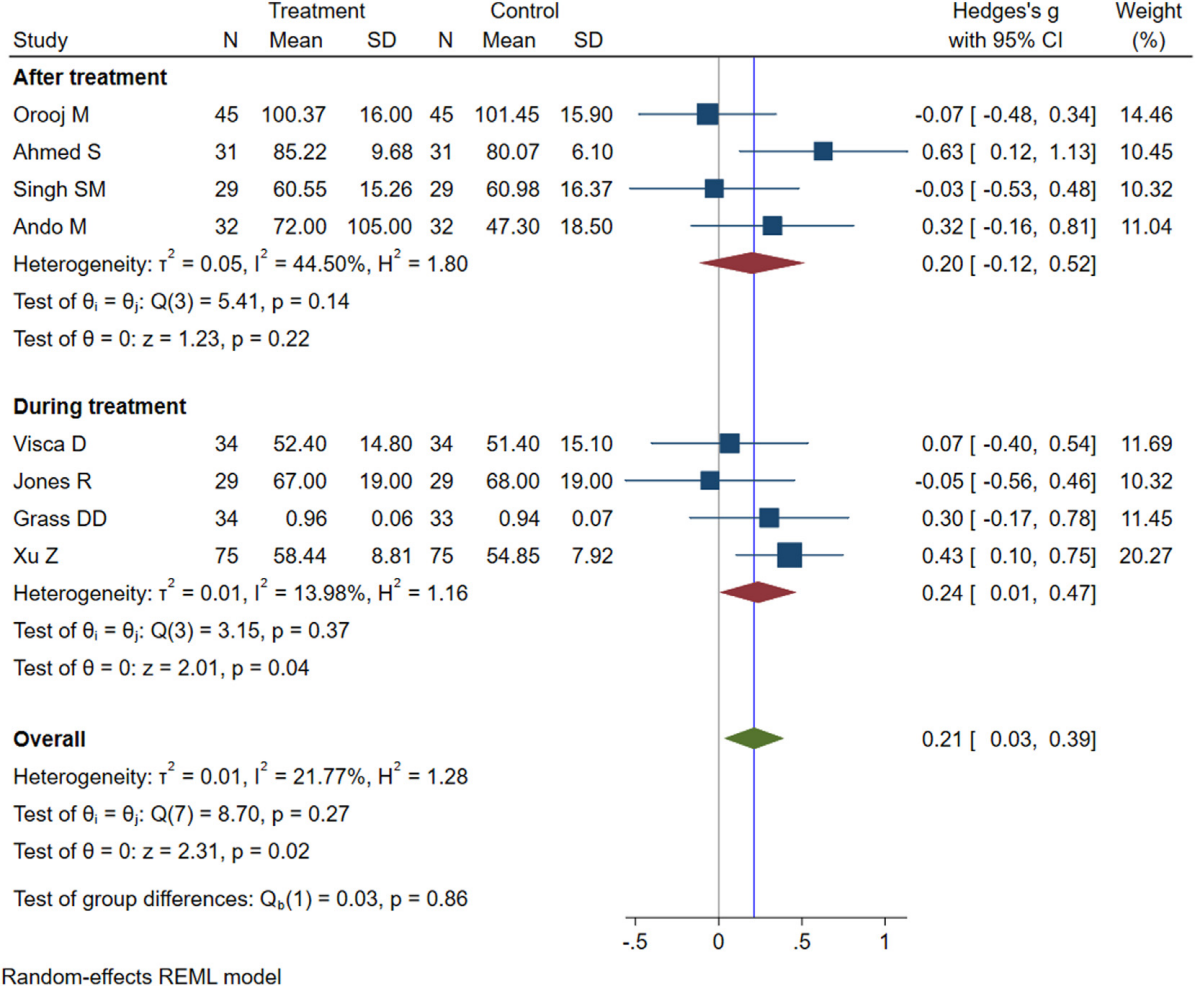
Forest plot representing the effects of interventions during and after treatment on preventing post-tuberculosis lung impairment.

Table 3 summarises interventions and their effectiveness in preventing post-TB lung sequelae.^18^, ^19^, ^20^, ^21^, ^22^, ^23^, ^24^, ^25^, ^26^ Nine studies were conducted to examine the effectiveness of various intervention strategies in mitigating post-TB lung function sequelae. These interventions include pulmonary rehabilitation programs, comprehensive nursing care and adjunctive surgical resection. Post-TB lung function was assessed using various tools, including a 6-min walk test, a daily activity score, clinical dyspnea rating, and spirometry measurements such as forced expiratory volume in 1 s (FEV1), forced vital capacity (FVC), and the ratio of FEV1 to FVC (FEV1/FVC). Notably, the FEV1/FVC ratio was commonly used across all studies (except one) to evaluate the impact of pulmonary rehabilitation programs on post-TB lung sequelae. We categorized interventions based on timing as those implemented during TB treatment and interventions implemented after the completion of TB treatment. Overall pooled effect estimates and subgroup analyses according to the timing of intervention are provided in Fig. 2. The pooled result shows that pulmonary rehabilitation programs can increase the grade of FEV1/FVC Ratio (Hedges's g = 0.21; 95% CI, 0.03–0.39; I^2^ = 21.7%; P = 0.02). Subgroup analyses by the timing of intervention showed that pulmonary rehabilitation programs provided during TB treatment significantly improved pulmonary function (Hedges's g = 0.24; 95% CI, 0.01–0.47; I^2^ = 13.98%; P = 0.04), whereas pulmonary rehabilitation programs provided after TB treatment did not significantly increase pulmonary function (Hedges's g = 0.20; 95% CI, −0.12 to 0.52; I^2^ = 44.5%; P = 0.22). The interaction test assessing the influence of pulmonary rehabilitation programs in relation to timing (during or after TB treatment) yielded non-significant results (Coefficient = 0.02, P = 0.8). This implies that the impact of the intervention is likely consistent across both time periods. Hower, it is important to interpret the findings cautiously, considering the small number of studies included in the meta-analysis and the lack of statistical significance does not necessarily imply the absence of a clinically meaningful difference. One study examining the effects of adjunctive surgical resection on pulmonary function showed that adjunctive surgical resection can improve pulmonary function. Results from sensitivity analyses, conducted using fixed-effects meta-analyses, were consistent with the values obtained from the random-effects meta-analysis (Figure S1). Analysis of funnel plots showed no obvious evidence of publication bias or that results of smaller trials were systematically different from those of larger trials (Figure S2). Main changes in lung function tests, pre- and post-intervention are available in Table S10.

**Table 3:** Studies reporting possible interventions for preventing post-tuberculosis lung function impairment and their main findings.

First author (year of publication), country, and study design	Category of intervention	Included interventions	Number of participants	Main findings
Ando M (2003), Japan, RCT	Pulmonary Rehabilitation	A 9-week pulmonary rehabilitation program	32	PRP appears to have merit when it is given to patients with post-TBC lung disorders
Grass DD (2014), South Africa, RCT	Pulmonary rehabilitation	A 6-week home-based pulmonary rehabilitation program that included cardiovascular exercises and walking.	67	PRP showed increasing trends towards lung function, but there was no statistical difference between the intervention and control groups
Jones R (2017), Uganda, RCT	Pulmonary rehabilitation	A 6-week pulmonary rehabilitation program consisted of exercise and education.	34	PRP was feasible and associated with clinically important improvements in respiratory outcomes, exercise capacity, and quality of life
Singh SM (2018), India, Prospective cohort	Pulmonary rehabilitation	An 8-week pulmonary rehabilitation program that included exercise and education.	29	Pulmonary function tests trended towards improvement with pulmonary rehabilitation but were not statistically significant
Vashakidze SA (2019), USA, Cross-sectional	Adjunctive surgical resection	Adjunctive lung surgery	54	Adjunctive surgery was significantly associated with improved lung function test
Xu Z (2021), China, RCT	Comprehensive nursing care	Comprehensive nursing intervention combined with respiratory functional exercises	150	Comprehensive nursing intervention combined with respiratory functional exercises can significantly improve pulmonary function
Orooj M (2023), India, RCT	pulmonary rehabilitation	A 4-week pulmonary rehabilitation program comprised of supervised exercise and education	90	Short-term PRP was insufficient to register changes in pulmonary function.
Ahmed S (2020), India, RCT	Pulmonary rehabilitation	12-week pulmonary rehabilitation program consisted of exercise training, breath retraining, and education.	62	PRP administered along with treatment improved lung function capacity.
Visca D (2019), Italy, Retrospective cohort	Pulmonary rehabilitation	3-week pulmonary rehabilitation programme including nurse training, exercises, relaxation, psychological support and nutritional counselling	43	PRP was effective in improving lung function capacity.

### Interventions to prevent drug-induced liver injury

**Fig. 3:**
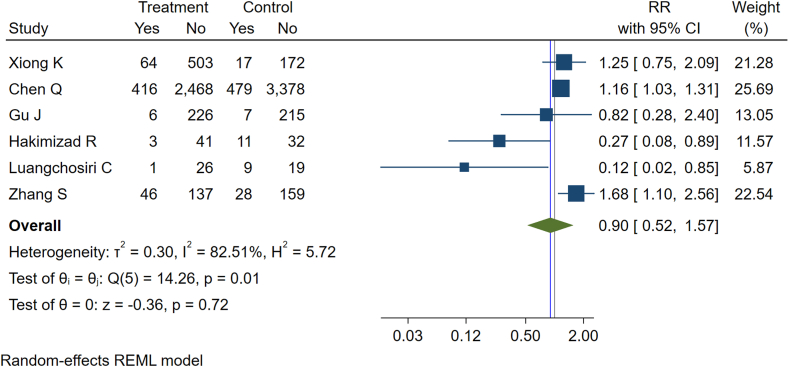
Forest plot showing the effect of intervention vs control in preventing post-tuberculosis liver function impairment.

Six studies reported the effectiveness of various interventions in preventing DILI that commonly leads to liver function sequelae (Table 4).^27^, ^28^, ^29^, ^30^, ^31^, ^32^ The interventions investigated included micronutrient supplementation (e.g., vitamin A and vitamin D), administration of mitochondrial nutrients (e.g., acetyl-l-carnitine, alpha-lipoic acid, and coenzyme Q10), and the use of hepatoprotective agents (such as silymarin, glycyrrhetinic acid, silibinin, and silybum marianum). The overall incidence rate of DILI was 128 per 1000 TB survivors. When comparing the intervention groups to control groups across six studies included in the meta-analysis, the pooled relative risk (RR) was 0.90 (95% CI: 0.52–1.57, P = 0.72), indicating a lack of statistical significance and, therefore, a lack of evidence that existing interventions effectively reduced the incidence of DILI (Fig. 3). Subgroup analyses based on intervention characteristics were unfeasible due to the limited number of studies available. However, when examining individual studies, it was found that two trials involving silymarin and mitochondrial nutrients demonstrated a significant reduction in liver injury, while the remaining four studies did not show a significant decrease in liver injury (Table 4). Publication bias was not observed in the funnel plot of studies included in the prevention of liver function impairment (Figure S3).

**Table 4:** Studies reporting possible interventions for preventing post-tuberculosis liver function impairment and their main findings.

First author (year of publication), country, and study design	Category of intervention	Included interventions	Number of participants	Main findings
Xiong K (2021), China, RCT	Vitamin A and Vitamin D	Vitamin A (2000 IU/d), vitamin D (400 IU/d), and combination of vitamins A & D (2000 IU/d) supplementation	753	Vitamin A and D supplementation did not protect against TB-drug-induced liver injury.
Chen Q (2022), China, Retrospective cohort	Hepatoprotection prophylactic therapies	Chemoprotective agents including silymarin and/or glycyrrhetinic acid during for the first 30 days	6743	Prophylactic utilisation of hepatoprotective agents was not associated with a reduction in TB-DILI risks
Gu J (2015), China, RCT	Preventive hepatoprotective therapy	Silibinin capsules (oral administration of 70 mg/time, 3 times/day for 8 weeks)	568	There was no statistical difference in the incidences of liver injury between the two groups at different treatment periods
Hakimizad R (2021), Iran, RCT	Protective mitochondrial nutrients	Combination of acetyl-l-carnitine (250 mg), alpha-lipoic acid (250 mg), and coenzyme Q10 (200 mg) orally twice per day for 2 weeks as mitochondrial nutrients against anti-TB drug-induced liver injury	87	The incidence of DILI in the experimental group was significantly lower than that in the placebo group.
Luangchosiri C (2015), Thailand, RCT	Hepatoprotection	Silymarin (a traditional herbal drug extracted from milk thistle (Silybum marinums) seeds, has been used as a supplement remedy for hepatoprotection)	55	Silymarin reduced the incidence of antiTB-DILI.
Zhang S (2015), China, RCT	Hepatoprotective	*Silybum marianum* hepatoprotectants ((oral, 200 mg, twice a day)) to prevent anti-tuberculosis drug-induced liver injury (ATLI).	370	No significant preventive effect of silymarin was found for either lowering the risk of liver injury or boosting the positive outcomes. Worse, it has found a potential risk of liver damage caused by the hepatoprotectant.

### Intervention to prevent neurological sequelae

**Fig. 4:**
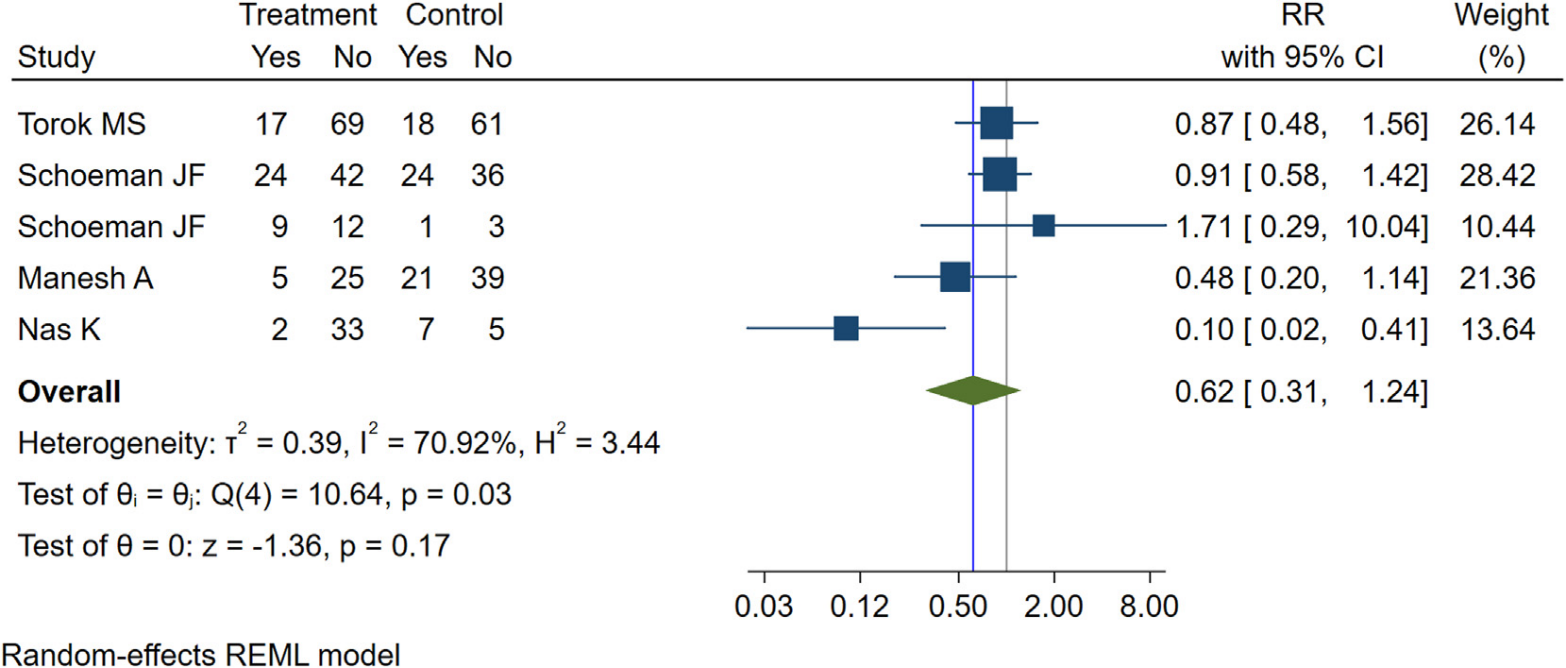
Forest plot showing the effect of intervention vs control in preventing post-tuberculosis neurological impairment.

There were 5 studies representing 806 participants that evaluated the impacts of interventions in preventing post-TB neurological sequelae such as neuromotor disability, hemiparesis, quadriparesis, and neurological deficits (Table 5).^33^, ^34^, ^35^, ^36^, ^37^ The overall incidence rate of neurological sequelae was 282 per 1000 TB survivors (239 per 1000 TB survivors in the intervention group and 330 per 1000 TB survivors in the control group). We identified two main components of interventions delivered in the study: adjunctive therapy and rehabilitation programs. Adjunctive therapy includes dexamethasone, prednisone, thalidomide, and infliximab, which were reported as a component of TB treatment in four studies. The results of the meta-analysis for these adjunctive therapies showed that there was no statistically significant difference in the incidence of post-TB neurological sequelae between the intervention and control groups (RR = 0.62, 95% CI: 0.31–1.24) (Fig. 4). No significant heterogeneity was observed among the studies (I^2^ = 70.92%, P = 0.17%). The sensitivity analyses using fixed-effects meta-analyses yielded similar results (Figure S4). In one study implementing rehabilitation programs, a significant reduction in the incidence of neurological sequelae was observed within the intervention group when compared to the control group (Table 5). Inspection of the funnel plot did not indicate publication bias (Figure S5).

**Table 5:** Studies reporting possible interventions for preventing post-tuberculosis neurological function impairment and their main findings.

First author (year of publication), country, and study design	Type of neurological function impairment	Category of intervention	Included interventions	Number of participants	Main findings
Nas K (2004), Turkey, Prospective cohort	Motor impairment	Rehabilitation program	Muscle-strengthening exercises postoperative for 6 months	47	Rehabilitation programs on motor and functional improve patients motor and functional development of patients with spinal tuberculosis.
Torok MS (2011), Vietnam, RCT	Neurological disability	Adjunctive dexamethasone therapy	Intravenous dexamethasone (0.3–0.4 mg/kg day) at presentation and tapered over six to eight weeks	545	Adjunctive treatment with dexamethasone improves patient survival with TB meningitis but probably does not prevent severe disability
Schoeman JF (1997), South Africa, RCT	Hemiparesis and quadriparesis	Adjuvant steroids therapy	High-dose prednisone as adjuvant steroids (i.e., corticosteroids) therapy	117	No significant difference was found in the incidence of motor deficit between the steroid and nonsteroid groups after the completion of treatment
Schoeman JF (2003), South Africa, RCT	Hemiparesis	Adjunctive Thalidomide therapy	Thalidomide (24 mg/kg/day orally) adjunctive therapy for one month	47	The results do not support the use of adjunctive high-dose thalidomide therapy in the treatment of TB meningitis. The motor outcome was similar in the intervention and control groups
Manesh A (2023), India, Retrospective cohort	Neurological deficits (severe disability)	Adjunctive Infliximab Therapy	Adjunctive High-Dose Infliximab Therapy (Cohort A received at least 1 dose of infliximab after optimal anti-TB treatment and steroids)	50	Infliximab may be an effective and safe adjunctive strategy among severely disabled patients with CNS TB.

### Intervention to prevent mental health disorders

**Fig. 5:**
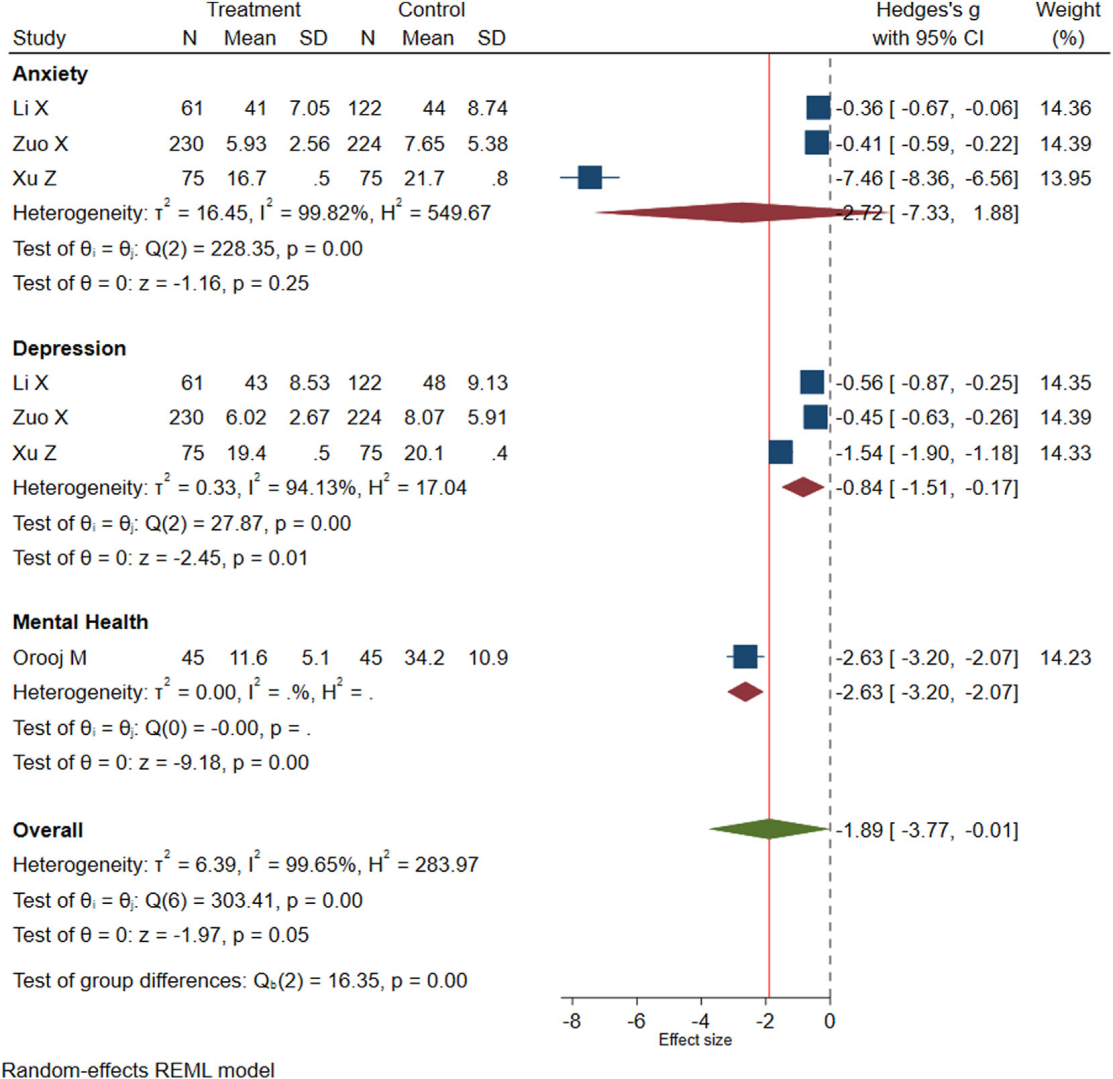
Forest plot representing the effect of intervention in preventing post-tuberculosis mental health disorders including anxiety and depression.

Four studies investigated the role of different interventions in preventing post-TB mental health disorders.^25^^,^^26^^,^^38^^,^^39^ Three of the studies assessed depression and anxiety as separate outcomes, while one study reported mental health as a general outcome (Table 6). We identified three different interventions reported in these studies for preventing the risk of post-TB mental health disorders, which included comprehensive interventions, pulmonary rehabilitation programs and cognitive-behavioural therapy (Table 6). These interventions significantly reduced the risk of mental health disorders among TB survivors (Hedges's g = −1.89; 95% CI: −3.77 to −0.01) (Fig. 5). However, there was publication bias (Figure S6) and the heterogeneity between studies was substantial (I^2^ = 99.65%, P < 0.001), with one study identified as an outlier among studies investigating the outcome of anxiety (Fig. 4). In contrast to the other studies that used standardised tools for anxiety measurement, the outlier study used a health-related quality of life questionnaire to assess anxiety. Sensitivity analyses were performed by excluding the outlier study, and the result showed a statistically significant reduction in anxiety within the intervention group compared to the control group (Hedges's g = −0.40; 95% CI: −0.56 to −0.24) (Figure S7).

**Table 6:** Studies reporting possible interventions for preventing post-tuberculosis mental health disorders impairment and their main findings.

First author (year of publication), country, and study design	Mental health disorder	Category of intervention	Included interventions	Number of participants	Main findings
Li X (2019), China, RCT	Depression and anxiety	Comprehensive interventions	Comprehensive interventions (health education, psychotherapy, home visits, peer support, and psycho-educational workshops)	183	Comprehensive interventions could alleviate the anxiety and depression of elderly tuberculosis patients sustainably and effectively.
Xu Z (2021), China, RCT	Depression and anxiety	Comprehensive interventions	Comprehensive nursing intervention combined with respiratory functional exercises	150	Comprehensive nursing intervention combined with respiratory functional exercises can significantly improve the pulmonary function, self-care ability, and quality of life of patients with pulmonary tuberculosis, with obvious clinical efficacy
Orooj M (2023), India, RCT	Mental health	Pulmonary rehabilitation program	Pulmonary rehabilitation program (comprised of supervised endurance and resistance training, breathing techniques, self-management strategies, and patient education)	90	A short-term pulmonary rehabilitation program enhances TB patient functional capacity, HRQoL, and dyspneal scores.
Zuo X (2022), China, RCT	Depression and anxiety	Cognitive-behavioral therapy	Cognitive-behavioral therapy for two months. The intervention group underwent CBT for 2 months, whereas the control group received routine follow-up	45	CBT can relieve anxiety, and depression symptoms and increase the quality of life in subjects with pulmonary tuberculosis.

### Intervention to prevent other types of post-TB sequelae

Table 7 presents summary information of four studies investigating interventions for post-TB cardiac function, hearing and vision sequelae.^33^^,^^40^, ^41^, ^42^ Adjuvant steroid therapy (i.e., prednisone) demonstrated a significant reduction in post-TB cardiac function sequelae while showing no preventive effect on hearing and visual sequelae.^33^^,^^41^ These results suggest that the effectiveness of prednisolone and other interventions may vary based on the specific type of sequelae and affected body sites. Additionally, one study reported the potential effects of early medical interventions including dose reduction, discontinuation of toxic medications, and regimen adjustments with safer drugs, in preventing post-TB hearing sequelae.^42^

**Table 7:** Studies reporting possible interventions for preventing other post-tuberculosis sequelae and their main findings.

First author (year of publication), country, and study design	Type of post-tuberculosis sequelae	Category of intervention	Included interventions	Number of participants	Main findings
Strange JIG (1988), South Africa, RCT	Abnormal cardiac function	Adjuvant steroids therapy	Adjuvant steroids therapy (i.e., prednisolone 5 mg tablets for first 11 weeks)	240	Prednisolone significantly reduced post-TB treatment sequala and corticosteroids can be prescribed in addition to anti-TB chemotherapy in the treatment of TB pericarditis.
Shangase KK (2019), South Africa, Retrospective	Hearing impairment	Early medical intervention	Early medical intervention to reduce the dose or stop treatment with ototoxic drugs, replaced by other drugs such as bedaquiline.	86	Early medical strategies implemented had a significant preventive impact.
Girgis NI (1983), Egypt, RCT	Vision impairment	Adjuvant steroids therapy	Adjunct therapy of dexamethasone was given (intramuscular 8–12 mg/day) for the first three weeks of therapy.	55	Dexamethasone does not affect the overall mortality from TBM but appears to prevent the development of severe ocular complications.
Schoeman JF (1997), South Africa, RCT	Vision and hearing impairment	Adjuvant steroids therapy	High-dose prednisone as adjuvant steroids (i.e., corticosteroids) therapy	117	No significant difference was found in the incidence of blindness or deafness between the steroid and nonsteroid groups.

## Discussion

This systematic review and meta-analysis quantifies the impacts of various interventions for preventing post-TB sequelae across different domains, including lung function impairment, liver function impairment, neurological impairment, and mental health disorders. These are the most common types of post-TB sequelae reported in previous systematic reviews and meta-analyses.^2^^,^^6^ In the current systematic review, several classes of interventions such as pulmonary rehabilitation programs, adjunctive surgical resection, comprehensive nursing care, micronutrient supplementation, adjunctive therapy, cognitive-behavioural therapy, hepatoprotective agents, and close patient follow-up and monitoring for timely medical intervention (e.g., dose reduction, treatment cessation, or switching to less toxic alternatives) were evaluated for their effectiveness in preventing different types of post-TB sequelae. The results demonstrated that only a few of these interventions have effectively prevented post-TB sequelae, providing important evidence for informed decision-making in managing post-TB sequelae. The scarcity of effective interventions to prevent and reduce post-TB sequelae may be attributed to the omission of post-TB sequelae from the TB program in the End TB Strategy. Furthermore, the recent UN New York declaration has also overlooked the emerging challenges of post-TB sequelae.

Our systematic review identified pulmonary rehabilitation programs as the most important intervention to prevent post-TB pulmonary sequelae among TB survivors. This finding aligns with previous systematic reviews that have also identified pulmonary rehabilitation programs as effective interventions in preventing sequelae in patients with chronic obstructive pulmonary disease (COPD).^43^^,^^44^ The studies included in our review presented diverse models of pulmonary rehabilitation programs, reflecting variations in healthcare contexts and access to such services. The rehabilitation programmes typically included physical exercises, strength training and education. Our study demonstrated that early initiation of the intervention may result in better lung function compared to late initiation; however, the evidence was not conclusive. This finding is clinically relevant and introduces a novel perspective on the significance of intervention timing in the context of TB care to optimise post-TB lung health. While integrating rehabilitation programs with TB treatment is important, further study is required to identify individuals most at risk for post-TB lung health complications. By targeting these high-risk groups, healthcare providers can ensure that pulmonary rehabilitation programs are delivered where they are needed most, thus maximising their potential impact to prevent post-TB sequelae. Here, it is also important to mention that early diagnosis and appropriate treatment of TB are crucial in preventing post-TB pulmonary sequelae by halting disease progression, minimizing lung tissue damage, preventing drug resistance, and reducing the risk of TB complications.^4^^,^^45^ A recent review showed that vaccination against respiratory tract infections including influenza, pneumococcal and COVID-19 potentially prevent post-TB lung sequelae.^46^ The Brazilian Thoracic Association has also recommended the use of these vaccinations to prevent post-TB lung sequelae.^47^

While the extent of liver function impairment among TB survivors may vary based on the treatment regimen, our systematic review examined potential interventions to prevent liver function impairment following TB treatment. Various interventions, including micronutrient supplementation, administration of mitochondrial nutrients, and the use of hepatoprotective agents, were evaluated in the existing literature to assess their effectiveness in preventing liver injury in patients with TB treatment. However, our meta-analysis did not reveal a statistically significant reduction in liver function impairment across the included studies, suggesting that there is a lack of evidence supporting their effectiveness. However, the low number of patients and methodological quality should be considered as a limitation when interpreting this finding. Previous studies also reported that while hepatoprotective agents were effective in preventing temporary and mild side effects of TB medications, they are not effective in preventing severe and long-term liver function impairment.^48^^,^^49^

Few studies have investigated the effects of rehabilitation programs and adjunctive therapy on preventing post-TB neurological impairment and findings are inconsistent.^33^, ^34^, ^35^, ^36^, ^37^ The pooled meta-analysis of adjunctive therapy revealed no statistically significant difference in neurological impairment incidence between the intervention and control groups, whereas a separate study on rehabilitation programs showed a significant reduction in neurological impairment within the intervention group compared to controls. While these findings suggest the potential benefit of rehabilitation programs in preventing neurological sequelae following TB treatment, further studies are needed to establish conclusive evidence.

Our systematic review identified four randomised controlled trials examining the effect of various interventions to prevent the occurrence of post-TB mental health disorders such as anxiety and depression.^25^^,^^26^^,^^38^^,^^39^ The interventions were based on comprehensive care, pulmonary rehabilitation programs, and cognitive-behavioural therapy. Meta-analysis of the included studies demonstrated that the interventions were considerably more effective than the control group (i.e., routine care or no intervention) in preventing post-TB mental health disorders. This has important implications for the longer-term management of depression and anxiety within TB programs. Interventions to prevent mental health disorders during TB illness have been studied extensively and the WHO guidelines have also recommended the integration of mental health programs during TB treatment.^50^ However, our findings showed that long-term integration of programs beyond the treatment period is also required.

The findings of this systematic review and meta-analysis need to be interpreted in the context of the following limitations. First, our results for most of the interventions are based on a limited number of studies. For example, while there were studies investigating the effectiveness of interventions for preventing post-TB cardiac impairment,^41^ hearing impairment,^33^^,^^42^ and vision impairments,^33^^,^^40^ conducting a meta-analysis was not possible due to the small number of studies. Second, as our systematic review and meta-analysis focused on identifying effective interventions for preventing long-term sequelae of TB or its medication, several studies evaluating the impacts of interventions in preventing temporary side effects of TB medication or disease complications were excluded. This conservative approach might miss some studies that did not separately report post-TB sequelae. Third, the comprehensive inclusion of diverse studies covering various topics, including pulmonary and extrapulmonary TB, and drug-sensitive and drug-resistant TB, in our systematic review and meta-analysis, poses a challenge in maintaining a cohesive narrative and may obscure the clarity of key findings. Fourth, the evidence in this systematic review is also not sufficiently robust to properly assess the effect of each intervention on the outcome of interest. This is primarily due to the small sample size and the very limited data in the existing literature, which is an important knowledge gap. Finally, our search was limited to English language studies, and this might have resulted in missing studies published in other languages.

In conclusion, this systematic review and meta-analysis identified various interventions for the prevention of post-TB sequelae. The results showed the importance of timing and specific interventions in addressing different sequelae. Rehabilitation programs show promise in preventing post-TB sequelae, while adjuvant therapies including hepato-protective drugs require further investigation. The varying quality of included studies and the presence of substantial heterogenicity suggest the need for adequately powered future studies using standardised endpoints. Clinicians and TB program leaders can use these findings to inform their strategies for preventing post-TB sequelae and improving the long-term health outcomes of TB patients.

## Contributors

KAA conceived and designed the study, ran the analysis, and drafted the manuscript. LH, BG, ACAC, and MBM made substantial contributions in reviewing the draft manuscript. All authors contributed to the final approval of the version to be submitted.

All authors had full access to all the data in the study and had final responsibility for the decision to submit for publication.

## Data sharing statement

The datasets generated during and/or analysed during the current study are available in the text and Supplementary material. Rough data supporting reported results are available from the corresponding author on reasonable request.

## Declaration of interests

The authors have no conflicts of interest to declare.
