# Supplementary material for: Interventions to prevent post-tuberculosis sequelae: a systematic review and meta-analysis
Source: eClinicalMedicine. 2024 Feb 26;70:102511. doi: 10.1016/j.eclinm.2024.102511 (PMC10907188; doi:10.1016/j.eclinm.2024.102511)
Supplement: Supplementary Figures and Tables [file mmc1.docx]

**Supplementary materials**

Interventions to prevent post-tuberculosis sequelae: a systematic review and meta-analysis

*Kefyalew Addis Alene, Lucas Hertzog, Beth Gilmour, Archie CA Clements, Megan B Murray*

Table of contents

[Figure S1: Forest plot representing the effects of interventions during and after treatment on preventing post-TB lung impairment based on a fixed effect inverse-variance model. 2](#_Toc158152982)

[Figure S2: Funnel plot for studies included in the prevention of lung function impairment. 3](#_Toc158152983)

[Figure S3: Funnel plot for studies included in the prevention of liver function impairment. 4](#_Toc158152984)

[Figure S4: Forest plot showing the effect of intervention versus control in preventing post-TB neurological impairment based on a fixed effect model. 4](#_Toc158152985)

[Figure S5: Funnel plot for studies included in the prevention of neurologic impairment. 5](#_Toc158152986)

[Figure S6: Funnel plot for studies included in the prevention of post-TB mental health disorders. 6](#_Toc158152987)

[Figure S7: Forest plot for the effect of intervention in preventing post-TB mental health disorders including anxiety and depression, after excluding outlier studies. 7](#_Toc158152988)

[Table S3: Search strategies for post-tuberculosis sequelae interventions in four different 8](#_Toc158152989)

[databases (PubMed, SCOPUS, ProQuest, and Web of Science). 8](#_Toc158152990)

[Table S4: Eligibility criteria for studies to be included in the systematic review based on PICOS (Population, Intervention, Control, Outcome and Study type). 10](#_Toc158152991)

[Table S5: Variables extracted from included studies. 11](#_Toc158152992)

[Table S6: Adjusted variables for the observational studies included in our review. 12](#_Toc158152993)

[Table S7: Studies excluded by full-text review and reason for exclusion. 13](#_Toc158152994)

[Table S8: Cochrane risk of bias assessment for RCTs 15](#_Toc158152995)

[Table 9: Newcastle Ottawa QA adapted for cross-sectional studies. 20](#_Toc158152996)

[Table S10: Main changes in lung function tests pre- and post-interventions 21](#_Toc158152997)

[References 21](#_Toc158152998)

Supplementary Figures

# Figure S1: Forest plot representing the effects of interventions during and after treatment on preventing post-TB lung impairment based on a fixed effect inverse-variance model.

# Figure S2: Funnel plot for studies included in the prevention of lung function impairment.

# Figure S3: Funnel plot for studies included in the prevention of liver function impairment.

# Figure S4: Forest plot showing the effect of intervention versus control in preventing post-TB neurological impairment based on a fixed effect model.

# Figure S5: Funnel plot for studies included in the prevention of neurologic impairment.

# Figure S6: Funnel plot for studies included in the prevention of post-TB mental health disorders.

# Figure S7: Forest plot for the effect of intervention in preventing post-TB mental health disorders including anxiety and depression, after excluding outlier studies.

Supplementary Tables

# Table S3: Search strategies for post-tuberculosis sequelae interventions in four different

# databases (PubMed, SCOPUS, ProQuest, and Web of Science).

|  | The search was made on 22 September 2023. | |
| --- | --- | --- |
| Search | Query | Results |
| PubMed | | |
| # 1 | ("tuberculosis"[MeSH] OR "mycobacterium tuberculosis"[MeSH] OR "tuberculosis"[Title/Abstract] OR "tuberculoses"[ Title/Abstract] OR "TB"[ Title/Abstract]) | 300,999 |
| # 2 | (sequelae[tiab] OR sequel[tiab] OR sequela*[tiab] OR disabil*[tiab] OR complicat*[tiab] OR consequence*[tiab] OR illness*[tiab] OR function*[tiab] OR chronic*[tiab] OR impair*[tiab] OR "disorder*"[tiab] OR failure*[tiab] OR loss*[tiab] OR "injury"[tiab] OR damage*[tiab] OR "lung function"[tiab] OR "lung failure"[tiab] OR "lung disease*" [tiab] OR pleura[tiab] OR "fibrosis"[tiab] OR "copd"[tiab] OR "chronic obstructive"[tiab] OR "bronchiectasis"[tiab] OR "pulmonary atrophy"[tiab] OR "lung atrophy"[tiab] OR "pulmonary disease*"[tiab] OR "respiratory impairment*"[tiab] OR "respiratory failure*"[tiab] OR "respiratory abnormal*"[tiab] OR "pulmonary impairment*"[tiab] OR "pulmonary failure* "[tiab] OR "pulmonary abnormal*"[tiab] OR "musculoskeletal impairment*"[tiab] OR "musculoskeletal failure*"[tiab] OR "musculoskeletal abnormal*"[tiab] OR "neurologic failure*"[tiab] OR " "neurologic impairment*" OR "neurologic abnormal *" OR "Pott’s disease"[tiab] OR "renal failure"[tiab] OR "renal impairment*"[tiab] OR "renal abnormal*"[tiab] OR "renal toxicity"[tiab] OR "nephrotoxicity"[tiab] OR "Liver failure"[tiab] OR "hepatic impairment*"[tiab] OR "hepatic failure*"[tiab] OR "hepatic abnormal*"[tiab] OR "cardiac impairment*"[tiab] OR "cardiac failure*"[tiab] OR "cardiac failure*"[tiab] OR "hearing impairment"[tiab] OR "hearing loss"[tiab] OR "hearing failure*"[tiab] OR "hearing abnormal*"[tiab] OR "deaf*"[tiab] OR ototoxicity[tiab] OR "vision loss" [tiab] OR visual impairment*"[tiab] OR "visual failure*"[tiab] OR "visual failure*"[tiab] OR "vision impairment*"[tiab] OR"vision failure*"[tiab] OR "vision abnormal*"[tiab] OR blindness[tiab] OR "blind*"[tiab] OR "mental health"[tiab] OR depression[tiab] OR anxiety[tiab] OR "developmental delay"[tiab] | 1,087,073 |
| #3 | (interven*[tiab] OR prevent*[tiab] OR avoid[tiab] OR control*[tiab] OR reduc*[tiab] OR eliminat*[tiab] OR prophyla* [tiab] OR program*[tiab] OR strategy* [tiab] OR treat*[tiab] OR therap*[tiab] OR rehab*[tiab] OR protect*[tiab]) | 15,681,867 |
| # | #3 OR 4 OR 5 OR 6 | 1,857 |
| Limit to | Human and English | 1,333 |
| SCOPUS | | |
| 1 | TITLE ("tuberculosis" OR "TB") | 177,867 |
| 2 | TITLE ( "sequelae" OR "sequel" OR "sequela*" OR "disabil*" OR "complicat*" OR "consequence*" OR "illness*" OR "function*" OR "chronic*" OR "impair*" OR "disorder*" OR "failure*" OR "loss*" OR "injury" OR damage* OR "disease*" OR "fibrosis" OR "copd" OR "bronchiectasis" OR "atrophy" OR "abnormal*" OR "toxicity" OR "nephrotoxicity" OR "deaf*" OR "ototoxicity" OR "blind*" OR "mental" OR depression OR anxiety OR "developmental delay" ) | 6,921,268 |
| 3 | TITLE ( "interven*" OR "prevent*" "OR avoid" OR "control*" OR "reduc*" OR "eliminat*” OR "prophyla*" OR "program*" OR "strategy*" OR "treat*" OR "therap*” OR "rehab*” OR "protect*" ) | 188,970 |
| 4 | # 1 AND #2 AND #3 | 176 |
| Web of Science | | |
| #1 | TI= ("Tuberculosis” OR "TB") | 121,981 |
| #2 | TI= ("sequelae" OR "sequel" OR "sequela*" OR "disabil*" OR "complicat*" OR "consequence*" OR "illness*" OR "function*" OR "chronic*" OR "impair*" OR "disorder*" OR "failure*" OR "loss*" OR "injury" OR damage* OR "disease*" OR "fibrosis" OR "copd" OR "bronchiectasis" OR "atrophy" OR "abnormal*" OR "toxicity" OR "nephrotoxicity" OR "deaf*" OR "ototoxicity" OR "blind*" OR "mental" OR depression OR anxiety OR "developmental delay") | 6,610,596 |
|  | TI= ("interven*" OR "prevent*" "OR avoid" OR "control*" OR "reduc*" OR "eliminat*” OR "prophyla*" OR "program*" OR "strategy*" OR "treat*" OR "therap*” OR "rehab*” OR "protect*") | 6,912,476 |
| #4 | #1 AND #2 AND #3 | 1,915 |
| Limit | English article | 1,079 |
| Cochrane Central | | |
| #1 | "tuberculosis" OR "TB" | 4739 |
| #2 | "sequelae" OR "sequel" OR "sequela" OR "disabil" OR "complicat" OR "consequence" OR "illness" OR "function" OR "chronic" OR "impair" OR "disorder" OR "failure" OR "loss" OR "injury" OR damage OR "disease" OR "fibrosis" OR "copd" OR "bronchiectasis" OR "atrophy" OR "abnormal" OR "toxicity" OR "nephrotoxicity" OR "deaf" OR "ototoxicity" OR "blind" OR "mental" OR "depression" OR "anxiety" OR "development" | 482754 |
| #3 | "interven" OR "prevent" OR "avoid" OR "control" OR "reduc" OR "eliminat" OR "prophyla" OR "program" OR "strategy" OR "treat" OR "therap" OR "rehab" OR "protect" | 494426 |
| #4 | #1 AND #2 AND #3 | 177 |

# Table S4: Eligibility criteria for studies to be included in the systematic review based on PICOS (Population, Intervention, Control, Outcome and Study type).

| PICOS elements | Inclusion criteria | Exclusion criteria |
| --- | --- | --- |
| Objectives | To provide comprehensive evidence on the effectiveness of existing interventions in preventing post-TB sequelae | |
| Population | Study conducted on adults or children with any type of TB. | Study conducted on animal or patients who do not have TB |
| Intervention | Study reporting any type of intervention for preventing post-TB sequelae | Study without an intervention |
| Control | Study with a control group such as placebo, different intervention, standard of care, baseline (before intervention) or no intervention group | Study without a control group |
| Outcome | Study with any type of post-TB sequela | Study which did not report any kind of post-TB sequelae |
| Study type | All interventional and observational studies including randomised control trials and cohort studies | Case series, case report, ecological studies, systematic review |

# Table S5: Variables extracted from included studies.

We collected information about the characteristics of the studies, characteristics of the participants, and outcomes of interest. We have also collected information on the type of interventions provided for the patients and the main findings of the study.

| Category | Variables |
| --- | --- |
| Characteristics of the studies | - The name of the first author - Year of publication - Country where the study was conducted. - Study setting, - Study design |
| Characteristics of the participants | - Study population, - Mean or median age - The proportion of participants who were male - Sample size - Number of study participants in the intervention and control groups - Type of TB and affected body sites, - Drug-resistant pattern - Type of medications and duration of treatments, - Comorbidities, including HIV and diabetes mellitus (DM) - Total follow-up period |
| Outcomes of interest and type of interventions | - Type of sequelae - Reporting times of the sequelae - Definition of the sequelae - Type of intervention - Timing of the intervention |

# Table S6: Adjusted variables for the observational studies included in our review.

| First author | Publication year | Country | Study design | Intervention | Control | Type of post-TB sequelae | Adjusted variables |
| --- | --- | --- | --- | --- | --- | --- | --- |
| Vashakidze SA | 2019 | USA | Cross-sectional | Surgical resection | No surgery | Lung function | Age, sex, BMI, Tobacco use, Alcohol use, Hepatitis C, prior TB, drug-resistant pattern |
| Nas K | 2004 | Turkey | Prospective cohort | Rehabilitation program | Before intervention | Neurologic | Not adjusted |
| Singh SM | 2018 | India | Prospective cohort | Pulmonary rehabilitation | Before intervention | Lung function | Not adjusted |
| Shangase KK | 2019 | South Africa | Retrospective | Early medical intervention | No intervention | Hearing | Not adjusted |
| Chen Q | 2022 | China | Retrospective cohort | Silymarin /glycyrrhetinic acid | No intervention | Liver function | Age, sex, TB diagnosis year, high-dimensional propensity score, baseline AST, ALP, and total bilirubin results,  and comorbidity burdens in the year before TB diagnosis. |
| Manesh A | 2023 | India | Retrospective cohort | Infliximab | No infliximab | Neurological | Age, Sex, HIV status, and drug-resistant pattern |
| Visca D | 2019 | Italy | Retrospective cohort | Pulmonary rehabilitation | No intervention | Lung function | Smoking and other comorbidities |

# Table S7: Studies excluded by full-text review and reason for exclusion.

| SN | First author | Publication Year | Reason for exclusion | Title |
| --- | --- | --- | --- | --- |
| 1 | Horsfall PA | 1969 | No quantitative data | The importance of adequate treatment and early diagnosis in prevention of disability in pulmonary tuberculosis |
| 2 | Sharma S | 2012 | Conference abstract | Randomized phase II trial of homeopathy to prevent post treatment impairment of pulmonary tuberculosis |
| 3 | Jager PD | 2002 | No intervention | Hearing loss and nephrotoxicity treatment in patients with long-term aminoglycoside tuberculosis |
| 4 | Chung KP | 2011 | No intervention | Trends and predictors of changes in pulmonary function after treatment for pulmonary tuberculosis |
| 5 | Thwaites GE | 2011 | Duplicate data | Dexamethasone for the Treatment of Tuberculous Meningitis in Adolescents and Adults |
| 6 | Seddon JA | 2013 | No intervention | Hearing loss in children treated for multidrug resistant tuberculosis |
| 7 | Adriztina I | 2014 | Other language | Hearing and Balance Impairment in Tuberculosis Patient with Category 1st and 2nd Antituberculosis Treatment |
| 8 | Sagwa EL | 2015 | No intervention | Comparing amikacin and kanamycininduced hearing loss in multidrug-resistant tuberculosis treatment under programmatic conditions in a Namibian retrospective cohort |
| 9 | Altena RV | 2017 | No intervention | Reduced Chance of Hearing Loss Associated with Therapeutic Drug Monitoring of Aminoglycosides in the Treatment of Multidrug-Resistant Tuberculosis |
| 10 | Chin TA | 2019 | No intervention | Chronic lung disease in adult recurrent tuberculosis survivors in Zimbabwe: a cohort study |
| 11 | Jatoi S | 2019 | No intervention | Standard factors predicting success of Non-invasive ventilation are useful in treating Patients with POST Tuberculosis sequel |
| 12 | Laxmeshwar C | 2019 | No intervention and no sequelae | Beyond ‘cure’ and ‘treatment success’: quality of life of patients with multidrug-resistant tuberculosis |
| 13 | Llorenc V | 2020 | No TB patients | Anti-tuberculous Treatment Itself Might Prevent Visual Impairment in Presumed Tuberculosis-Related Uveitis |
| 14 | Shangase KK | 2021 | No intervention | Impact of drug-resistant tuberculosis treatment on hearing function in South African adults: Bedaquiline versus kanamycin |
| 15 | Toorn RV | 2021 | No adequate information | Thalidomide Use for Complicated Central Nervous System Tuberculosis in Children: Insights from an Observational Cohort |
| 16 | Ahmed S | 2020 | Abstract only | Role of N-acetylcysteine (NAC) in preventing development of anti-tuberculosis therapy(ATT) induced liver injury in pulmonary tuberculosis(PTB) patients, a simple randomized single-blind clinical trial |
| 17 | Karak B | 1998 | Inadequate information | Corticosteroids in tuberculous meningitis |
| 17 | Mbala L | 2998 | Inadequate information | Is vitamin B6 supplementation of isoniazid therapy useful in childhood tuberculosis |
| 18 | Ralp AP | 2013 | Temporary side effects only | L-arginine and Vitamin D Adjunctive Therapies in Pulmonary Tuberculosis: A Randomised, Double-Blind, Placebo-Controlled Trial |
| 19 | Tandon R | 2021 | Inadequate information | Evaluation of Radiological Sequelae after Treatment Completion in New Cases of Pulmonary and Pleural Tuberculosis |
| 20 | Azuma J | 2013 | Temporary side effects only | NAT2 genotype guided regimen reduces isoniazid-induced liver injury and early treatment failure in the 6-month four-drug standard treatment of tuberculosis: A randomized controlled trial for pharmacogenetics-based therapy |
| 21 | Tandon R | 2021 | No intervention | Evaluation of radiological sequelae after treatment completion in new cases of pulmonary and pleural tuberculosis |

# Table S8: Cochrane risk of bias assessment for RCTs

|  |  |  | Ahmed, 2022 | De Grass 2014 | Girgis, 1983 | Gu, 2015 | Hakimizad, 2021 | Li, 2019 | Luangchosiri, 2015 | Orooj, 2023 | Schoeman, 1997 | Schoeman, 2004 | Strang, 1988 | Torok, 2011 | Xiong, 2021 | Xu, 2021 | Zhang, 2016 | Zuo, 2022 |
| --- | --- | --- | --- | --- | --- | --- | --- | --- | --- | --- | --- | --- | --- | --- | --- | --- | --- | --- |
| Domain 1 | Risk from randomization process | 1.1 | PY | PY | N | PY | PY | PY | Y | Y | PY | Y | PY | Y | Y | PY | Y | Y |
|  |  | 1.2 | PY | NI | PY | NI | Y | PY | Y | Y | PY | NI | NI | Y | NI | NI | Y | NI |
|  |  | 1.3 | PN | PN | N | PN | PN | PN | PN | Y | N | PN | PN | PN | PN | PN | PY | PN |
|  |  | Judgement | LOW | LOW | LOW | LOW | LOW | LOW | LOW | LOW | LOW | LOW | LOW | LOW | LOW | LOW | SC | LOW |
|  |  | Direction | NA | NA | UP | NA | NA | NA | NA | NA | NA | NA | NA | NA | NA | NA | UP | NA |
| Domain 2 | Effect of assignment to intervention | 2.1 | PN | PY | PN | PN | N | PY | N | NA | PN | PN | NI | N | NI | NI | PN | PY |
|  |  | 2.2 | PY | PY | NI | NI | N | PY | N | PN | PN | PN | NI | N | NI | NI | NI | PY |
|  |  | 2.3 | PN | PN | NA | NA | NA | PN | NA | NA | NA | NA | NA | NA | NA | NA | NA | PN |
|  |  | 2.4 | NA | NA | NA | NA | NA | NA | NA | NA | NA | NA | NA | NA | NA | NA | NA | NA |
|  |  | 2.5 | NA | NA | NA | NA | NA | NA | NA | NA | NA | NA | NA | NA | NA | NA | NA | NA |
|  |  | 2.6 | PY | PY | PY | PY | PY | PY | PY | PY | PY | PY | PY | PY | PY | PY | Y | PY |
|  |  | 2.7 | NA | NA | NA | NA | NA | NA | NA | NA | NA | NA | NA | NA | NA | NA | NA | NA |
|  |  | Judgement | LOW | LOW | LOW | LOW | LOW | LOW | LOW | LOW | LOW | LOW | LOW | LOW | LOW | LOW | LOW | LOW |
|  |  | Direction | NA | NA | NA | NA | NA | NA | NA | NA | NA | NA | NA | NA | NA | NA | NA | NA |
| Domain 3 | Missing data outcome | 3.1 | Y | PN | Y | PY | PY | PY | Y | Y | Y | N | PY | PY | Y | PY | Y | Y |
|  |  | 3.2 | NA | PY | NA | NA | NA | NA | NA | NA | NA | PN | NA | NA | NA | NA | NA | NA |
|  |  | 3.3 | NA | NA | NA | NA | NA | NA | NA | NA | NA | NI | NA | NA | NA | NA | NA | NA |
|  |  | 3.4 | NA | NA | NA | NA | NA | NA | NA | NA | NA | NI | NA | NA | NA | NA | NA | NA |
|  |  | Judgement | LOW | LOW | LOW | LOW | LOW | LOW | LOW | LOW | LOW | SC | LOW | LOW | LOW | LOW | LOW | LOW |
|  |  | Direction | NA | NA | NA | NA | NA | NA | NA | NA | NA | UP | NA | NA | NA | NA | NA | NA |
| Domain 4 | Measurement of the outcome | 4.1 | PN | PN | PN | PN | PN | PN | PN | PN | N | PN | PN | PY | PN | PN | N | PN |
|  |  | 4.2 | PN | PN | PN | PN | PN | PN | N | N | N | PN | PN | PN | PN | PN | PN | PN |
|  |  | 4.3 | NI | N | NI | NI | N | N | N | NI | N | NI | NI | PY | NI | NI | NI | NA |
|  |  | 4.4 | PN | PN | NA | NA | NA | NA | NA | NA | NA | NA | NA | PN | NA | NA | NA | NA |
|  |  | 4.5 | NA | NA | NA | NA | NA | NA | NA | NA | NA | NA | NA | NA | NA | NA | NA | NA |
|  |  | Judgement | LOW | LOW | LOW | LOW | LOW | LOW | LOW | LOW | LOW | LOW | LOW | SC | LOW | LOW | LOW | LOW |
|  |  | Direction | NA | NA | NA | NA | NA | NA | NA | NA | NA | NA | NA | UP | NA | NA | NA | NA |
| Domain 5 | Selection of the reported result | 5.1 | PY | NI | NI | NI | NI | NI | NI | NI | NI | NI | NI | NI | NI | NI | NI | NI |
|  |  | 5.2 | PN | PN | PN | PN | PN | PN | N | PN | N | PN | PN | PN | PN | PN | N | PN |
|  |  | 5.3 | PN | PN | PN | PN | PN | PN | N | PN | N | PN | PN | PN | PN | PN | N | PN |
|  |  | Judgement | LOW | LOW | LOW | LOW | LOW | LOW | LOW | LOW | LOW | LOW | LOW | LOW | LOW | LOW | LOW | LOW |
|  |  | Direction | NA | NA | NA | NA | NA | NA | NA | NA | NA | NA | NA | NA | NA | NA | NA | NA |
| Overall risk of bias | | Judgement | LOW | LOW | LOW | LOW | LOW | LOW | LOW | LOW | LOW | SC | LOW | SC | LOW | LOW | SC | LOW |
|  |  | Direction | NA | NA | NA | NA | NA | NA | NA | NA | NA | UP | NA | UP | NA | NA | UP | NA |

Key to signalling questions:

- 1. Was the allocation sequence random?
  2. Was the allocation sequence concealed until participants were enrolled and assigned interventions?
  3. Did baseline differences between intervention groups suggest a problem with the randomisation process?

2.1 Were participants aware of their assigned intervention during the trial?

2.2 Were carers and people delivering the interventions aware of participants’ assigned intervention during the trial?

2.3 If Y/PY/NI to 2.1 or 2.2 Were there deviations from the intended intervention that arose because of the trial context?

2.4 If Y?PY to 2.3 Were these deviations likely to have affected the outcome?

2.5 If Y/PY/NI to 2.4 Were these deviations from intended intervention balance between groups?

2.6 Was an appropriate analysis used to estimate the effect of assignment to intervention?

2.7 If N/PN/NI to 2.6 Was there potential for a substantial impact (on the result) of the failure to analyse participants in the group to which they were randomized?

3.1 Were data from this outcome available for all, or nearly all, participants randomised?

3.2 If N/PN/NI to 3.1 Is there evidence that the result was not bias by missing outcome data?

3.3 If N/PN to 3.2 Could missingness in the outcome depend on its true value?

3.4 If Y/PY/NI to 3.3 Is it likely that missingness in the outcome depended on its true value?

4.1 Was the method of measuring the outcome appropriate?

4.2 Could measurement or ascertainment of the outcome have differed between intervention groups?

4.3 If N/PN/NI to 4.1 and 4.2 Were outcome assessors aware of the intervention received by study participants?

4.4 If Y/PY/NI to 4.3 Could assessment of the outcome have been influenced by knowledge of intervention received?

4.5 If Y/PY/NI to 4.4 Is it likely that assessment of the outcome was influenced by knowledge of intervention received?

5.1 Were the data that produced this result analysed in accordance with a pre-specified analysis plan that was finalised before unblinded outcome data were available for analysis?

Is the numerical result being assessed likely to have been selected on the basis of results from…….

5.2 …. multiple eligible outcome measurements (e.g., scales, definitions, time points) within the outcome domain?

5.3 …. Multiple eligible analyses of the data?

Key to responses:

N=no; NA= not applicable; NI= no information; PN= probably no; PY=probably yes; SC= some concerns; UP= unpredictable; Y=yes

Cochrane risk of bias assessment for cohort studies

|  |  | Ando, 1988 | Chen, 2022 | Jones, 2017 | Khoza-Shangase, 2020 | Manesh, 2023 | Nas, 2004 | Singh, 2018 | Visca, 2019 |
| --- | --- | --- | --- | --- | --- | --- | --- | --- | --- |
| Was the selection of exposed and non-exposed cohorts drawn from the same population? | Definitely yes (low risk of bias) |  | √ | √ |  |  |  | √ | √ |
|  | Probably yes |  |  |  | √ |  |  |  |  |
|  | Probably no |  |  |  |  |  |  |  |  |
|  | Definitely no (high risk of bias) | √ |  |  |  | √ | √ |  |  |
| Can we be confident in the assessment of exposure? | Definitely yes (low risk of bias) |  | √ |  |  |  |  |  |  |
|  | Probably yes | √ |  | √ | √ | √ | √ | √ | √ |
|  | Probably no |  |  |  |  |  |  |  |  |
|  | Definitely no (high risk of bias) |  |  |  |  |  |  |  |  |
| Can we be confident that the outcome of interest was not present at the start of the study | Definitely yes (low risk of bias) |  | √ |  |  |  |  |  |  |
|  | Probably yes | √ |  | √ | √ | √ | √ | √ | √ |
|  | Probably no |  |  |  |  |  |  |  |  |
|  | Definitely no (high risk of bias) |  |  |  |  |  |  |  |  |
| Did the study match exposed and unexposed for all variables that are associated with the outcome of interest or did the statistical analysis adjust for these prognostic variables? | Definitely yes (low risk of bias) | √ |  |  |  |  |  |  |  |
|  | Probably yes |  | √ | √ | √ |  | √ | √ | √ |
|  | Probably no |  |  |  |  | √ |  |  |  |
|  | Definitely no (high risk of bias) |  |  |  |  |  |  |  |  |
| Can we be confident in the assessment of the presence or absence of prognostic factors? | Definitely yes (low risk of bias) |  |  |  |  |  |  |  |  |
|  | Probably yes | √ | √ | √ | √ | √ | √ | √ | √ |
|  | Probably no |  |  |  |  |  |  |  |  |
|  | Definitely no (high risk of bias) |  |  |  |  |  |  |  |  |
| Can we be confident in the assessment of outcome? | Definitely yes (low risk of bias) |  |  |  |  |  |  |  | √ |
|  | Probably yes | √ | √ |  | √ | √ | √ | √ |  |
|  | Probably no |  |  | √ |  |  |  |  |  |
|  | Definitely no (high risk of bias) |  |  |  |  |  |  |  |  |
| Was the follow up of cohorts adequate? | Definitely yes (low risk of bias) |  |  |  |  |  |  |  |  |
|  | Probably yes | √ | √ | √ | √ | √ | √ | √ | √ |
|  | Probably no |  |  |  |  |  |  |  |  |
|  | Definitely no (high risk of bias) |  |  |  |  |  |  |  |  |
| Were interventions similar between groups? | Definitely yes (low risk of bias) |  |  | √ |  |  | √ | √ | √ |
|  | Probably yes | √ | √ |  | √ | √ |  |  |  |
|  | Probably no |  |  |  |  |  |  |  |  |
|  | Definitely no (high risk of bias) |  |  |  |  |  |  |  |  |
|  | RISK OF BIAS | HIGH | LOW | SC* | LOW | HIGH | HIGH | LOW | LOW |

Key: *SC= some concerns

# Table 9: Newcastle Ottawa QA adapted for cross-sectional studies.

| Vashakidze, 2019 | |
| --- | --- |
| SELECTION (max score= 5) |  |
| Representativeness of the cases:  a) Truly representative of TB patients (consecutive or random sampling of cases). 1 score  b) Somewhat representative of the average in TB patients (non-random sampling) . 1 score  c) Selected demographic group of patients. 0 score  d) No description of the sampling strategy. 0 score | 1 |
| Sample size:  a) Justified and satisfactory 1 score  b) Not justified 0 score | 0 |
| Non-Response rate a) The response rate is satisfactory (≥95%). 1 Score  b) The response rate is unsatisfactory (<95%) 0 score | 0 |
| Ascertainment of the screening/surveillance tool:  a) Validated screening/surveillance tool. 2 scores  b) Non-validated screening/surveillance tool, but the tool is available or described. 1 score  c) No description of the measurement tool. 0 score | 1 |
| COMPARABILITY : (maximum score=1 )  The potential confounders were investigated by subgroup analysis or multivariable analysis.  a) The study investigates potential confounders. 1 score  b) The study does not investigate potential confounders. 0 score | 0 |
| OUTCOME: (maximum score= 3 ) |  |
| Assessment of the outcome:  a) Independent blind assessment. 2 scores  b) Record linkage. 2 scores  c) Self report. 1 score  d) No description. 0 score | 2 |
| Statistical test:  a) The statistical test used to analyse the data is clearly described and appropriate. 1 score  b) The statistical test is not appropriate, not described or incomplete. 0 score | 1 |

# Table S10: Main changes in lung function tests pre- and post-interventions

| Studies | Pre-intervention | Post-intervention | Changes in lung function test | Measurements |
| --- | --- | --- | --- | --- |
| Orooj M; 2023 | 313.00 | 484.15 | 171.15 | Six-minute walk distance in meter |
| Xu Z; 2021 | 54.85 | 58.44 | 3.59 | FEV1/FVC |
| Ahmed S; 2020 | 80.07 | 85.22 | 5.15 | FEV1/FVC |
| Vashakidze SA; 2019 | 71.00 | 72.00 | 1.00 | FEV1/FVC |
| Visca D; 2019 | 51.40 | 52.40 | 1.00 | FEV1/FVC |
| Singh SM; 2018 | 488.00 | 526.00 | 38.00 | Six-minute walk distance in meter |
| Jones R; 2017 | 312.41 | 402.07 | 89.66 | Six-minute walk distance in meter |
| Grass DD; 2014 | 94.00 | 96.00 | 2.00 | FEV1/FVC |
| Ando M; 2003 | 47.30 | 72.00 | 24.70 | Vital capacity |
